# Compatibility of family and clinical–scientific career for German gynecologists in different workplaces: a sub-analysis of the systematic trinational FARBEN survey

**DOI:** 10.1007/s00404-025-08087-3

**Published:** 2025-08-29

**Authors:** Niklas Amann, Maggie Banys-Paluchowski, Claudia Becker, Philipp Foessleitner, Martin Göpfert, Rama Kiblawi, Nora Kiessling, Amanda Klee, Natalia Krawczyk, Laura Dussan Molinos, Gert Naumann, Achim Rody, Henning Schäffler, Lina Schiestl, Barbara Schmalfeldt, Solveig Simowitsch, Martin Weiss, Nikolas Tauber

**Affiliations:** 1 Department of Gynecology and Obstetrics, University Hospital Erlangen, Comprehensive Cancer Center Erlangen-European Metropolitan Region of Nuremberg, Erlangen, Germany; 2 Department of Gynecology and Obstetrics, University Hospital Schleswig-Holstein, Campus Luebeck, Luebeck, Germany; 3 Department of Gynecology and Obstetrics, Winterthur Cantonal Hospital, Winterthur, Switzerland; 4 Department of Obstetrics and Gynecology, Division of Obstetrics and Feto-Maternal Medicine, Medical University of Vienna, Vienna, Austria; 5 Kinderwunsch Centrum Muenchen, Munich, Germany; 6 University Hospital Basel Women’s Clinic, Basle, Switzerland; 7 Department of Obstetrics and Gynecology, Vivantes Auguste-Viktoria Clinic, Berlin, Germany; 8 Department of Obstetrics and Gynecology, Hietzing Clinic, Vienna, Austria; 9 Department of Gynecology and Obstetrics, University Hospital Duesseldorf, 40225 Duesseldorf, Germany; 10 Oberschwabenklinik Ravensburg, Ravensburg, Germany; 11 Department of Obstetrics and Gynaecology, Helios-Klinikum, Erfurt, Germany; 12 Department of Gynecology and Obstetrics, University Hospital Ulm, Ulm, Germany; 13 Department of Obstetrics and Gynecology, university medical center, Johannes Gutenberg University Mainz, Mainz, Germany; 14 Department of Gynecology, University Medical Center Hamburg-Eppendorf, Hamburg, Germany; 15 University of Luebeck, Luebeck, Germany; 16 Department of Gynecology and Obstetrics, University of Tuebingen, Tuebingen, Germany

**Keywords:** Working time models, University hospital, Career goals, Gender differences, Childcare

## Abstract

**Introduction:**

The trinational survey project conducted by the young forums of the German, Austrian, and Swiss societies for gynecology and obstetrics aims to evaluate the preferences of prospective and practicing gynecologists regarding various working time models, training systems, career pathways, and the reconciliation of professional and family life.

**Materials and methods:**

Between October 2023 and May 2024, 1364 participants completed the FARBEN survey. The questionnaire comprised 62 items addressing aspects such as general workplace conditions, working time models, training priorities, team structures, and professional aspirations. Participation was voluntary and anonymous. The present analysis constitutes a national sub-analysis focusing on the preferences of German respondents, stratified by their current workplace setting (university hospitals, non-university hospitals, or outpatient care facilities).

**Results:**

Among the 1008 German respondents, 26.3% were employed in university hospitals, 55.4% in non-university hospitals, and 10.8% in outpatient care. Respondents working in university hospitals were significantly more likely to pursue an academic career (68.0% held a doctoral degree, and 7.5% held a habilitation—the highest academic qualification in German-speaking countries—or a professorship; 38.5% aspired to a habilitation, compared to 6.6% in non-university hospitals). Female respondents employed in university hospitals tended to have children at a later stage and returned to work earlier following parental leave. Institutional childcare was reported to be more accessible and flexible at university hospitals (20.4%) compared to non-university hospitals (9.6%) and outpatient care (8.4%). Furthermore, 34.1% of university hospital respondents indicated that their department head was female, in contrast to 19.2% in non-university hospitals (*p* < 0.001).

**Conclusions:**

Work–life balance and the compatibility of family life with a medical career are essential for most gynecologists in training, irrespective of their workplace setting. Respondents employed in academic institutions were more inclined to pursue scientific careers and reported greater flexibility and better childcare support. In light of the growing shortage of medical professionals, employment policies should prioritize these aspects. Initiatives such as the guideline “Safe Surgery During Pregnancy” can support the alignment of clinical training with family planning and help prevent career disadvantages related to parenthood.

## What does this study add to the clinical work

**Table:** 

The FARBEN study represents the first systematic assessment of working time models among physicians in gynecology within German-speaking countries, with a specific focus on residents. In light of the current shortage of qualified healthcare professionals, such data are essential for identifying structural deficits and informing targeted strategies to improve clinical working conditions. During residency, decisions made by young physicians are strongly influenced by considerations related to family planning and career development. This sub-analysis aimed to compare university and non-university hospitals in this context.	
The results indicate that the availability and flexibility of employer-supported childcare are significantly more favorable in university hospitals. Additionally, respondents working in university hospitals were more frequently under the leadership of female department heads than their counterparts in non-university settings.	
Given the persistent workforce shortages in the healthcare sector, there is an urgent need to implement comprehensive support measures for both family life and academic advancement. In particular, the expansion of family-friendly working conditions—especially for female physicians—is critical to enable the compatibility of a demanding clinical-academic career with personal and family responsibilities.	

## Introduction

In 2023, 428,474 physicians were employed in Germany [1]. While internal medicine remains the most frequently chosen specialty with 61,899 physicians, gynecology and obstetrics also represent a substantial share, with 19,530 specialists [1]. Compared to other medical disciplines, gynecology and obstetrics exhibit a particularly high proportion of female physicians. As of 2023, 14,268 specialists in this field were women, placing it second only to internal and general medicine in terms of female representation [1].

In contrast to many other countries, the majority of medical care in Germany is delivered by physicians working in outpatient settings. Approximately, two–thirds of the gynecologists practice in outpatient care, while the remaining one-third work in inpatient settings [1]. Despite the predominance of women in the specialty, male physicians are disproportionately represented in leadership positions within hospitals. Of 822 chief physicians in the field, only 244 are women [1]. Conversely, in outpatient care, 6166 of 8591 private practice owners are female [1].

Inpatient gynecological care in Germany is delivered through university hospitals, large non-university tertiary care centers, and specialized non-university hospitals [2, 3]. In general, specialty training in gynecology and obstetrics spans 5–6 years, with a minimum of 3 years of inpatient hospital training required for board certification [4]. Physicians independently apply to institutions for residency training and receive financial remuneration during the course of their specialty education [4]. University hospitals are distinguished by their dual role in providing medical care and engaging residents in undergraduate medical teaching, academic training, and research activities [5].

The evolving expectations of society regarding work–life dynamics have had a considerable impact on the medical profession [6]. As an increasing number of women pursue medical degrees and enter the workforce as physicians, the compatibility of career and family life has gained prominence as a decisive factor [7, 8]. Working hours and the availability of childcare can be critical determinants in the choice of employer [9].

In response to these developments, the young forum of the German society for gynecology and obstetrics (DGGG e.V.), in collaboration with the young gynecology group of the Austrian society for gynecology and obstetrics (OEGGG) and the young forum of the Swiss society for gynecology and obstetrics (SGGG), launched a trinational survey focusing on individual preferences in key areas such as working time models, professional goals, and family leave—the FARBEN survey [9, 10].

The present study represents a sub-analysis of the FARBEN survey. Its aim is to compare preferences concerning working hours, clinical and academic goals, research engagement, and work–family compatibility among gynecologists and obstetricians in different employment settings—university hospitals, non-university hospitals, and outpatient care—and to identify potential differences as well as their underlying causes.

## Methods

Between October 2023 and May 2024, 1364 participants completed the online survey, which was initiated by the young forums of the German, Austrian, and Swiss societies for gynecology and obstetrics, in collaboration with the University of Lübeck (FARBEN [German: colors]: FAvorisierte aRBEitszeitmodelle in der Gynäkologie = Preferred Working Time Models in Gynecology). Participation was anonymous.

## Questionnaire

The questionnaire has been described in detail elsewhere [9]. In brief, it was developed through a consensus process by representatives of the young boards and colleges of obstetrics and gynecology of Germany, Austria, and Switzerland (NT, NA, PF, AK, CB, RK), under the mentorship of Prof. Dr. Maggie Banys-Paluchowski (University Hospital Schleswig–Holstein, Lübeck Campus) and the Gender Equality Officer of the University of Lübeck, Dr. Solveig Simowitsch. The instrument was a self-developed, non-validated questionnaire. In addition, Prof. Dr. Barbara Schmalfeldt, President of the German board and college of obstetrics and gynecology (GBCOG), provided advisory support.

The trilingual questionnaire was created using the online platform SurveyMonkey and consisted of 62 questions. It was available in German, Italian, and French, allowing participants to respond in their preferred native language. Of these 62 questions, 54 (see Appendix) were presented to all participants, regardless of their country of origin. In addition, one country-specific question was posed to German participants, three to Austrian participants, and four to Swiss participants, focusing on national training structures and healthcare systems. Three of the total 62 questions were designed as free-text responses. Ethical approval was obtained from the ethics committee of the University of Lübeck (File number: 2023–644). The phrasing of the questions was adapted to reflect the specific healthcare and training environments of each participating country.

### Recruitment and survey invitations

Participants were recruited through various channels, including the social media platforms (primarily Instagram) of the respective young forums and young gynecology groups, print media, educational events and conferences, newsletters of national gynecological societies, and lectures for medical students [10]. The target group encompassed medical students interested in gynecology and obstetrics, residents, board-certified specialists, senior physicians, department heads, and gynecologists working in outpatient care.

### Subgroup analysis

As part of the FARBEN study, a national subgroup analysis was conducted based on current workplace settings. For this analysis, only participants from Germany who were employed in university/non-university departments, or outpatient care settings were included. Participants from Austria and Switzerland were excluded. After identifying the relevant subgroup, the statistical analysis was performed.

### Statistical analysis

Data were analyzed using microsoft excel version 2311 and IBM SPSS statistics version 29.0.2.0 (Armonk, NY: IBM Corp.). Duplicate entries were excluded through anonymous IP address screening. Associations between categorical variables were assessed using the Chi-square test, with *p* values < 0.05 considered statistically significant. All *p* values reported are two-sided.

## Results

### Participant characteristics

Of the 1008 participants from Germany, 91.3% identified as female, 8.5% as male, and 0.2% as diverse (Table 1). Overall, 65.4% were residents in specialty training, 16.2% were board-certified specialists, 12.3% were senior physicians, 1.2% held positions as chief physicians, 4.8% were gynecologists practicing in outpatient settings, and 8.9% were medical students. The latter group was excluded from further analyses.

**Table 1:** Analysis of the questions on the topic: demographics and professional objectives categorized by workplace

Questions	Total*: *n* (%)	Workplace		
		University department	Non-university department	Outpatient care
**Your workplace**	**1008 (100.0)**			
University department	265 (26.3)			
Non-university women’s department with a total of > 25 physicians	176 (17.5)			
Non-university women’s department with a total of 15–25 physicians	243 (24.1)			
Non-university women’s department with a total of < 15 physicians	139 (13.8)			
Outpatient care/MVZ (employed)	73 (7.2)			
Outpatient care/MVZ (self-employed)	36 (3.6)			
Student	60 (6.0)			
Other (combination of outpatient and department, outpatient care in Austria, etc.)	16 (1.6)			
**In which federal state do you work/study?**	**932 (100.0)**	**265 (100.0)**	**558 (59.9)**	**109 (11.7)**
Baden-Württemberg	115 (12.3)	38 (14.3)	61 (10.9)	16 (14.7)
Bavaria	183 (19.6)	69 (26.0)	101 (18.1)	13 (11.9)
Berlin	59 (6.3)	9 (3.4)	40 (7.2)	10 (9.2)
Brandenburg	24 (2.6)	4 (1.5)	20 (3.6)	0 (0.0)
Bremen	13 (1.4)	0 (0.0)	11 (2.0)	2 (1.8)
Hamburg	68 (7.3)	14 (5.3)	44 (7.9)	10 (9.2)
Hesse	61 (6.5)	6 (2.3)	53 (9.5)	2 (1.8)
Mecklenburg Western Pomerania	22 (2.4)	4 (1.5)	12 (2.2)	6 (5.5)
Lower Saxony	54 (5.8)	6 (2.3)	40 (7.2)	8 (7.3)
North Rhine–Westphalia	159 (17.1)	55 (20.8)	89 (15.9)	15 (13.8)
Rhineland Palatinate	31 (3.3)	14 (5.3)	16 (2.9)	1 (0.9)
Saarland	5 (0.5)	1 (0.4)	3 (0.5)	1 (0.9)
Saxony-Anhalt	12 (1.3)	2 (0.8)	8 (1.4)	2 (1.8)
Saxony	33 (3.5)	9 (3.4)	23 (4.1)	1 (0.9)
Schleswig–Holstein	87 (9.3)	31 (11.7)	34 (6.1)	22 (20.2)
Thuringia	6 (0.6)	3 (1.1)	3 (0.5)	0 (0.0)
**What is your sex?**				
Female	851 (91.3)	221 (83.4)	528 (94.6)	102 (93.6)
Male	79 (8.5)	44 (16.6)	28 (5.0)	7 (6.4)
Diverse	2 (0.2)	0 (0.0)	2 (0.4)	0 (0.0)
**How old are you?**	**932 (100.0)**	**265 (28.4)**	**558 (59.9)**	**109 (11.7)**
18–20	0 (0.0)			
21–30	287 (30.8)	108 (40.8)	177 (31.7)	2 (0.2)
31–40	503 (54.0)	133 (50.2)	311 (55.7)	59 (54.1)
41–50	96 (10.3)	16 (6.0)	45 (8.1)	35 (32.1)
51–60	36 (3.9)	7 (2.6)	20 (3.6)	9 (8.3)
61–70	8 (0.9)	0 (0.0)	4 (0.7)	4 (3.7)
71–older	2 (0.2)	1 (0.4)	1 (0.2)	0 (0.0)
**What is your current further education/professional position?**	**909 (100.0)**	**248 (27.3)**	**542 (59.6)**	**119 (13.1)**
Resident 1 st year	76 (8.3)	27 (10.9)	48 (8.9)	1 (0.8)
Resident 2nd year	76 (8.3)	17 (6.9)	58 (10.7)	1 (0.8)
Resident 3rd year	129 (14.2)	43 (17.3)	83 (15.3)	3 (2.5)
Resident 4th year	122 (13.4)	29 (11.7)	85 (15.7)	8 (6.7)
Resident 5th year	133 (14.6)	36 (14.5)	81 (14.9)	16 (13.4)
Resident 6th year	42 (4.6)	6 (2.4)	28 (5.2)	8 (6.7)
Resident > 6th year	18 (2.0)	4 (1.6)	13 (2.4)	1 (0.8)
Specialist physician	147 (16.2)	47 (19.0)	63 (11.6)	37 (31.1)
Senior physician	112 (12.3)	35 (14.1)	77 (14.2)	0 (0.0)
Chief physician	10 (1.1)	4 (1.6)	6 (1.1)	0 (0.0)
Gynecologist in outpatient care	44 (4.8)	0 (0.0)	0 (0.0)	44 (37.0)
**Your professional goal is this position:**	**932 (100.0)**	**265 (28.4)**	**558 (59.9)**	**109 (11.7)**
Chief physician	50 (5.4)	33 (12.5)	17 (3.0)	0 (0.0)
(Leading) senior physician	349 (37.4)	135 (50.9)	211 (37.8)	3 (2.8)
Employed medical specialist in the department	98 (10.5)	27 (10.2)	71 (12.7)	0 (0.0)
Employed in outpatient care	151 (16.2)	11 (4.2)	99 (17.7)	41 (37.6)
Self-employed in outpatient care	227 (24.4)	42 (15.8)	125 (22.4)	60 (55.0)
Other (please specify)	57 (6.1)	17 (6.4)	35 (6.3)	5 (4.6)

*The differing number of total responses per question is due to the fact that participants were able to skip questions or prematurely end the survey

**Fig. 1:**
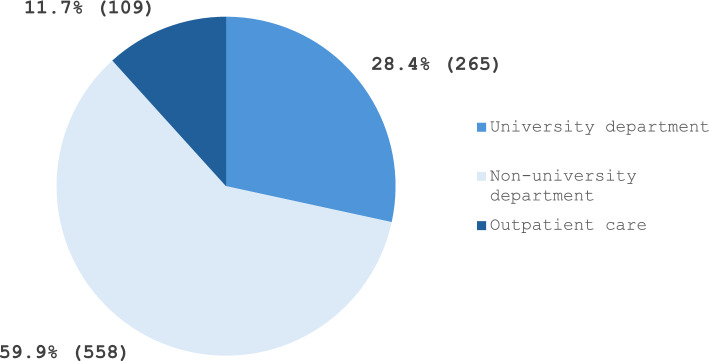
Workplaces of survey participants employed in Germany and included in the present subgroup analysis

Regarding current workplace settings, 28.4% of respondents were employed in university departments, 59.9% in non-university departments, and 11.7% in outpatient care (including MVZ—Medizinisches Versorgungszentrum, i.e., ambulatory healthcare centers) (see Fig. 1). Among respondents from non-university departments, 43.5% reported working in departments with 15–25 physicians, 31.5% in departments with more than 25 physicians, and 24.9% in smaller teams with fewer than 15 physicians. In total, 84.8% of participants were between 21 and 40 years of age (Table 2).

**Table 2:** Analysis of the questions on the topic: family planning categorized by workplace

Questions	Total*: *n* (%)	Workplace			*p* value
		University department	Non-university department	Outpatient care	
**Do you have children?**	**932 (100.0)**	**265 (28.4)**	**558 (59.9)**	**109 (11.7)**	** < 0.001**
Yes	466 (50.0)	92 (34.7)	284 (50.9)	90 (82.6)	
No	466 (50.0)	173 (65.3)	274 (49.1)	19 (17.4	
**With your first child you were:**^**1)**^	**466 (100.0)**	**92 (19.7)**	**284 (60.9)**	**90 (19.3)**	**0.003**
Student	77 (16.6)	15 (16.3)	48 (16.9)	14 (15.5)	
Senior physician	33 (7.1)	7 (7.6)	23 (8.1)	3 (3.3)	
Chief physician	0 (0.0)	0 (0.0)	0 (0.0)	0 (0.0)	
Gynecologist in outpatient care	0 (0.0)	8 (0.0	0 (0.0)	0 (0.0	
**How long did you not work after giving birth? (For multiple children with different time off, please use multiple answers)**^**1)**^	**466 (100.0)**	**92 (19.7)**	**284 (60.9)**	**90 (19.3)**	** < 0.001**
2 years per child	28 (6.2)	3 (3.3)	17 (6.0)	8 (8.9)	
1–2 years per child	172 (38.0)	21 (22.8)	113 (39.8)	38 (42.2)	
9–12 months per child	195 (43.0)	32 (34.8)	123 (43.3)	40 (44.4)	
6–8 months per child	49 (10.8)	16 (17.4)	26 (9.2)	7 (7.8)	
3–5 months per child	18 (4.0)	9 (9.8)	4 (1.4)	5 (5.6)	
< 3 months per child	26 (5.7)	12 (13.0)	10 (3.5)	4 (4.4)	
You did not take any family leave	28 (6.2)	6 (6.5)	18 (6.3)	4 (4.4)	
Other option (please specify)	20 (4.3)	7 (7.6)	10 (3.5)	3 (3.3)	
**Do you want to become a parent in the future?**^**2)**^	**466 (100.0)**	**173 (37.1)**	**274 (58.8)**	**19 (4.1)**	** < 0.001**
Yes	383 (82.2)	145 (83.8)	231 (84.3)	7 (36.8)	
No	31 (6.7)	9 (5.2)	15 (5.5)	7 (36.8)	
You are not sure yet	52 (11.2)	19 (11.0)	28 (10.2)	5 (26.3)	
**How long would you like to pause work after the birth of your child as a parent?**^**2)**^	**435 (100.0)**	**164 (37.7)**	**259 (59.5)**	**12 (2.8)**	** < 0.001**
2 years per child	9 (2.1)	4 (2.4)	5 (1.9)	0 (0.0)	
1–2 years per child	110 (25.3)	28 (17.1)	80 (30.9)	2 (16.7)	
9–12 months per child	153 (35.2)	41 (25.0)	109 (42.1)	3 (25.0)	
6–8 months per child	72 (16.6)	42 (25.6)	28 (10.8)	2 (16.7)	
3–5 months per child	25 (5.7)	15 (9.1)	10 (3.9)	0 (0.0)	
< 3 months per child	9 (2.1)	6 (3.7)	3 (1.2)	0 (0.0)	
You do not want to take any family leave	2 (0.5)	2 (1.2)	0 (0.0)	0 (0.0)	
You are not sure yet	55 (12.6)	26 (15.9)	24 (9.3)	5 (41.7)	

*The differing number of total responses per question is due to the fact that participants were able to skip questions or prematurely end the survey

^1^This question was only directed at parents

^2^This question was only directed at childless people

The regional distribution of participants corresponded closely to the overall population distribution in Germany. Specifically, 19.6% of respondents resided in Bavaria, 17.1% in North Rhine-Westphalia, and 12.3% in Baden-Württemberg.

### Gender differences according to workplace

**Fig. 2:**
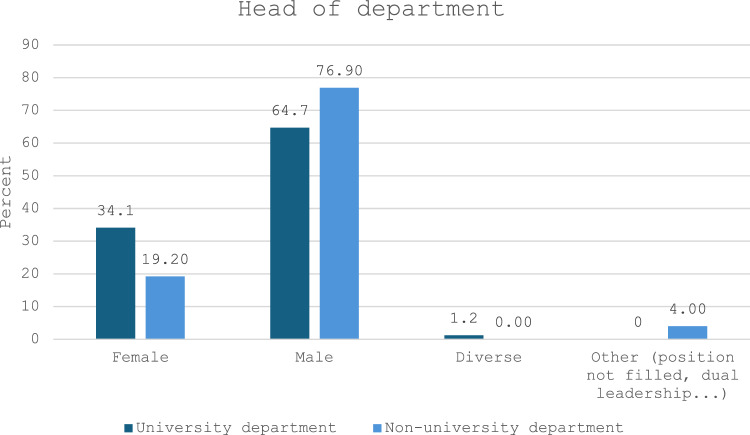
Gender distribution of heads of departments reported by survey participants employed in Germany

Gender distribution varied significantly between university and non-university departments (*p* < 0.001) (Table 3). Among participants employed at university departments, 21.6% reported that ≥ 40% of their colleagues were male, in contrast to only 3.1% of respondents from non-university departments. Likewise, 33.1% of participants from non-university departments indicated that there were no male residents in their team, compared to just 4.8% of respondents from university departments. Furthermore, participants from university departments were significantly more likely to report having a female head of department (34.1%) compared to those from non-university departments (19.2%, *p* < 0.001) (see Fig. 2) (Table 4).

**Table 3:** Analysis of the questions on the topic: medical staff categorized by workplace

Questions	Total*: *n* (%)	Workplace			*p* value
		University department	Non-university department	Outpatient care	
**How many male residents are there in your team?**^**1**^	**802 (100.0)**	**239 (31.0)**	**553 (69.0)**		** < 0.001**
> 50%	15 (1.9)	11 (4.4)	4 (0.7)		
Ca. 50%	28 (3.5)	22 (8.8)	6 (1.1)		
Ca. 40%	28 (3.5)	21 (8.4)	7 (1.3)		
Ca. 30%	73 (9.1)	45 (18.1)	28 (5.1)		
Ca. 20%	146 (18.2)	70 (28.1)	76 (13.7)		
Ca. 10%	317 (39.5)	68 (27.3)	249 (45.0)		
There is no male resident	195 (24.3)	12 (4.8)	183 (33.1)		
**Your department head is:**^**1**^	**816 (100.0)**	**249 (31.0)**	**553 (69.0)**		** < 0.001**
Female	191 (23.8)	85 (34.1)	106 (19.2)		
Male	586 (73.1)	161 (64.7)	425 (76.9)		
Diverse	3 (0.4)	3 (1.2)	0 (0.0)		
Other (position not filled, dual leadership, etc.)	22 (2.7)	0 (0.0)	22 (4.0)		
**What percentage of mothers in your department take family leave?**^**1**^	**802 (100.0)**	**249 (31.0)**	**553 (69.0)**		0.11
100– > 80%	591 (73.7)	177 (71.1)	414 (74.9)		
> 60–80%	54 (6.7)	26 (10.4)	28 (5.1)		
> 40–60%	28 (3.5)	11 (4.4)	17 (3.1)		
> 20–40%	19 (2.4)	6 (2.4)	13 (2.4)		
1–20%	19 (2.4)	7 (2.8)	12 (2.2)		
Mothers do not take family leave at your department	9 (1.1)	1 (0.4)	8 (1.4)		
You do not know	79 (9.9)	20 (8.0)	59 (10.7)		
There are no mothers at your department	3 (0.4)	1 (0.4)	2 (0.4)		
**What percentage of fathers in your department take family leave?**^**1**^	**802 (100.0)**	**249 (31.0)**	**553 (69.0)**		** < 0.001**
100– > 80%	79 (9.9)	28 (11.2)	51 (9.2)		
> 60–80%	68 (8.5)	45 (18.1)	23 (4.2)		
> 40–60%	69 (8.6)	31 (12.4)	38 (6.9)		
> 20–40%	80 (10.0)	39 (15.7)	41 (7.4)		
1–20%	130 (16.2)	44 (17.7)	86 (15.6)		
Fathers do not take family leave at your department	89 (11.1)	12 (4.8)	77 (13.9)		
You do not know	172 (21.4)	40 (16.1)	132 (23.9)		
There are no fathers at your department	115 (14.3)	10 (4.0)	105 (19.0)		
**Family leave at your workplace is supported:**	**879 (100.0)**	**236 (26.8)**	**539 (61.3)**	**104 (11.8)**	**0.037**
Do not agree at all	56 (6.4)	13 (5.5)	35 (6.5)	8 (7.7)	
Largely disagree	63 (7.2)	19 (8.1)	37 (6.9)	7 (6.7)	
Rather disagree	136 (15.5)	45 (19.1)	70 (13.0)	21 (20.2)	
Neither	181 (20.6)	49 (20.8)	105 (19.5)	27 (26.0)	
Rather agree	174 (19.8)	51 (21.6)	103 (19.1)	20 (19.2)	
Largely agree	188 (21.4)	42 (17.8)	127 (23.6)	19 (18.3)	
Strongly agree	81 (9.2)	17 (7.2)	62 (11.5)	2 (1.9)	
**Does your workplace have a concept for returning to work after a longer period of family leave (e.g., return to work with an initially reduced workload)?**	**849 (100.0)**	**227 (26.7)**	**525 (61.8)**	**97 (11.4)**	0.29
Yes, a re-entry concept is available	79 (9.3)	27 (11.9)	47 (9.0)	5 (5.2)	
No, I am not aware of the existence of such a concept. But I think it is desirable	671 (79.0)	172 (75.8)	421 (80.2)	78 (80.4)	
No, I am not aware of the existence of such a concept and do not consider it necessary	99 (11.7)	28 (12.3)	57 (10.9)	14 (14.4)	
**Does your place of work provide childcare with flexible hours and sufficient capacity?**	**842 (100.0)**	**225 (26.7)**	**522 (62.0)**	**95 (11.3)**	** < 0.001**
Yes	104 (12.4)	46 (20.4)	50 (9.6)	8 (8.4)	
No	616 (73.2)	130 (57.8)	408 (78.2)	78 (82.1)	
You do not know	122 (14.5)	49 (21.8)	64 (12.3)	9 (9.5)	

*The differing number of total responses per question is due to the fact that participants were able to skip questions or prematurely end the survey

^1^This question was only directed at participants who work in a hospital

**Table 4:** Analysis of the questions on the topic: Working-time models in the departments and medical practice categorized by workplace

Questions	Total*: *n* (%)	Workplace			*p* value
		University department	Non-university department	Outpatient care	
**Your preferred working time model is:**	**879 (100.0)**	**236 (26.8)**	**539 (61.3)**	**104 (11.8)**	** < 0.001**
100%	103 (11.7)	44 (18.6)	52 (9.6)	7 (6.7)	
90–99%	51 (5.8)	19 (8.1)	29 (5.4)	3 (2.9)	
80–89%	364 (41.4)	106 (44.9)	231 (42.9)	27 (26.0)	
70–79%	234 (26.6)	47 (19.9)	154 (28.6)	33 (31.7)	
60–69%	82 (9.3)	15 (6.4)	49 (9.1)	18 (17.3)	
50–59%	43 (4.9)	5 (2.1)	23 (4.3)	15 (14.4)	
< 50%	2 (0.2)	0 (0.0)	1 (0.2)	1 (1.0)	
**Your current working time model is:**	**879 (100.0)**	**236 (26.8)**	**539 (61.3)**	**104 (11.8)**	** < 0.001**
100%	461 (52.4)	181 (76.7)	254 (47.1)	26 (25.0)	
90–99%	23 (2.6)	1 (0.4)	14 (2.6)	8 (7.7)	
80–89%	129 (14.7)	13 (5.5)	105 (19.5)	11 (10.6)	
70–79%	69 (7.8)	12 (5.1)	44 (8.2)	13 (12.5)	
60–69%	62 (7.1)	9 (3.8)	42 (7.8)	11 (10.6)	
50–59%	51 (5.8)	4 (1.7)	19 (3.5)	28 (26.9)	
< 50%	13 (1.5)	2 (0.8)	6 (1.1)	5 (4.8)	
You are currently on maternity leave	24 (2.7)	3 (1.3)	20 (3.7)	1 (1.0)	
You are currently on family leave as a parent	35 (4.0)	11 (4.7)	23 (4.3)	1 (1.0)	
You are currently looking for work/not yet employed	12 (1.4)	0 (0.0)	12 (2.2)	0 (0.0)	
**Did you reduce your working hours after the birth of your child?**^**1**^	**344 (100.0)**	**41 (11.9)**	**228 (66.3)**	**75 (21.8)**	0.14
I have no children	72 (20.9	6 (14.6)	57 (25.0)	9 (12.0)	
Yes	249 (72.4)	32 (78.0)	156 (68.4)	61 (81.3)	
No	23 (6.7)	3 (7.3)	15 (6.6)	5 (6.7)	
**What solutions would you propose for avoiding a possible personnel burden due to part-time workers? (multiple answers possible)**^**2**^	**315 (100.0)**	**120 (38.1)**	**181 (57.5)**	**14 (4.4)**	**0.007**
Fixed job sharing (2 physicians each 50% with duty splitting)	154 (48.9)	52 (43.3)	98 (54.1)	4 (28.6)	
Part-time employees work exclusively full days with fixed days off	174 (55.2)	73 (60.8)	97 (53.6)	4 (28.6)	
I do not know	81 (25.7)	31 (25.8)	42 (23.2)	8 (57.1)	
Other (please specify)	29 (9.2)	11 (9.2)	18 (9.9)	0 (0.0)	
**The optimal time to become a parent is for you:**	**849 (100.0)**	**227 (26.7)**	**525 (61.8)**	**97 (11.4)**	0.87
During medical school	58 (5.8)	17 (7.5)	34 (6.5)	7 (7.2)	
During specialty training	169 (19.9)	36 (15.9)	112 (21.3)	21 (21.6)	
As medical specialist	187 (22.0)	55 (24.2)	109 (20.8)	23 (23.7)	
As senior (leading) physician	34 (4.0)	12 (5.3)	20 (3.8)	2 (2.1)	
As chief physician	1 (0.1)	0 (0.0)	1 (0.2)	0 (0.0)	
As a physician in outpatient care	5 (0.6)	1 (0.4)	3 (0.6)	1 (1.0)	
There is no optimal time	395 (46.5)	106 (46.7)	246 (46.9)	43 (44.3)	
**Can you imagine taking a sabbatical?**	**849 (100.0)**	**227 (26.7)**	**525 (61.8)**	**97 (11.4)**	**0.004**
You do not want to take a sabbatical	232 (27.3)	47 (20.7)	144 (27.4)	41 (42.3)	
Maximum 3 months	97 (11.4)	23 (10.1)	70 (13.3)	4 (4.1)	
3–6 months	228 (26.9)	73 (32.2)	133 (25.3)	22 (22.7)	
6–12 months	232 (27.3)	66 (29.1)	142 (27.0)	24 (24.7)	
More than 12 months	60 (7.1)	18 (7.9)	36 (6.9)	6 (6.2)	

*The differing number of total responses per question is due to the fact that participants were able to skip questions or prematurely end the survey

^1^This question was only directed at part-time workers

^2^This question was only directed at full-time workers

### The scientific–academic career in residency.

More than half of all respondents, irrespective of their current workplace, held a doctoral degree. The proportion of participants with an academic title (at least a doctorate) was highest among those working in outpatient care (75.8%) and university departments (75.5%—including doctoral degree: 68.0%, habilitation: 4.4% [the highest academic qualification in German-speaking countries], and professorship: 3.1%), compared to those in non-university departments (60.0%—including doctoral degree: 58.8%, habilitation: 0.2%, professorship: 1.0%) (*p* < 0.001) (Table 5). At non-university departments, 33.1% of respondents reported having no academic title, whereas this applied to only 19.1% of those working in university departments.

**Table 5:** Analysis of the questions on the topic: the scientific-academic career categorized by workplace

Questions	Total*: *n* (%)	Workplace			*p* value
		University department	Non-university department	Outpatient care	
**Your highest academic title is:**	**842 (100.0)**	**225 (26.7)**	**522 (62.0)**	**95 (11.3)**	** < 0.001**
Master’s degree^1^	51 (6.1)	12 (5.3)	36 (6.9)	3 (0.4)	
Doctoral degree	532 (63.2)	153 (68.0)	307 (58.8)	72 (75.8)	
Habilitation^3^	11 (1.3)	10 (4.4)	1 (0.2)	0 (0.0)	
Professorship	12 (1.4)	7 (3.1)	5 (1.0)	0 (0.0)	
You do not have an academic title	236 (28.0)	43 (19.1)	173 (33.1)	20 (21.1)	
**You would like to achieve habilitation**^**2)**^	**819 (100.0)**	**208 (25.4)**	**516 (63.0)**	**95 (11.6)**	** < 0.001**
Yes	115 (14.0)	80 (38.5)	34 (6.6)	1 (1.1)	
No	549 (67.0)	66 (31.7)	394 (76.4)	89 (93.7)	
You do not know yet	155 (18.9)	62 (29.8)	88 (17.1)	5 (5.3)	
**In your opinion, which parts of further training in gynecology and obstetrics cannot be adequately implemented due to family leave (multiple answers possible)?**	**830 (100.0)**	**225 (27.1)**	**512 (61.7)**	**93 (11.2)**	**0.006**
Obstetrics training	140 (16.9)	43 (19.1)	80 (15.6)	17 (18.3)	
Acquisition of special skills in gynecological diagnostics (e.g., breast sonography, colposcopy/dysplasia)	313 (37.7)	65 (28.9)	215 (42.0)	33 (35.5)	
Surgical training	605 (72.9)	150 (66.7)	390 (76.2)	65 (69.9)	
Care of inpatients	33 (4.0)	10 (4.4)	19 (3.7)	4 (4.3)	
Conservative management of gynecological clinical diseases	41 (4.9)	6 (2.7)	30 (5.9)	5 (5.4)	
Management of gynecological emergencies	77 (9.3)	24 (10.7)	46 (9.0)	7 (7.5)	
All parts of the training are adequately implemented despite family leave	207 (24.9)	67 (29.8)	114 (22.3)	26 (28.0)	
**In your opinion, which parts of specialty training in gynecology and obstetrics cannot be adequately implemented due to part-time work (multiple answers possible)**	**830 (100.0)**	**225 (27.1)**	**512 (61.7)**	**93 (11.2)**	**0.021**
Obstetric training	122 (14.7)	42 (18.7)	61 (11.9)	19 (20.4)	
Acquisition of special skills in gynecological diagnostics (e.g., breast sonography, dysplasia)	309 (37.2)	69 (30.7)	205 (40.0)	35 (37.6)	
Surgical training	572 (68.9)	149 (66.2)	352 (68.8)	71 (76.3)	
Care of inpatients	61 (7.3)	22 (9.8)	30 (5.9)	9 (9.7)	
Conservative management of gynecological diseases	27 (3.3)	6 (2.7)	16 (3.1)	5 (5.4)	
Management of gynecological emergencies	79 (9.5)	24 (10.7)	45 (8.8)	10 (10.8)	
All parts of the training are adequately implemented despite family leave	217 (26.1)	61 (27.1)	138 (27.0)	18 (19.4)	

*The differing number of total responses per question is due to the fact that participants were able to skip questions or prematurely end the survey

^1^Master Degree: additional academic title like master of health business administration

^2^This question was only directed at participants without habilitation

^3^Habilitation = highest university degree in German-speaking countries; only respondents without habilitation/professorship were asked this question

Future academic aspirations also differed notably between groups: 38.5% of participants from university departments indicated a desire to pursue a habilitation, compared to 6.6% of respondents from non-university departments and 1.1% from outpatient care (*p* < 0.001). When stratified by gender, 33.3% of women employed in university departments reported an ambition to complete a habilitation, compared to only 6.3% of their counterparts in non-university departments (*p* < 0.001). Among male respondents, 67.7% working at university departments aspired to a habilitation, in contrast to just 12.5% from non-university departments (*p* < 0.001).

Respondents employed at university departments were also significantly more likely to aspire to leadership roles compared to their non-university counterparts (*p* < 0.001). Specifically, 63.4% of university-based participants reported career ambitions toward leadership positions (12.5% aspiring to become head of department and 50.9% to become senior physicians), compared to 40.8% of respondents from non-university departments (3.0% aiming for head of department, 37.8% for senior physician).

In contrast, the proportion of respondents intending to transition into outpatient care in the future was highest among those currently employed in non-university departments (40.1%) compared to 20.0% of those in university departments. Regardless of the current workplace, the most frequently cited reasons for intending to leave the hospital setting were similar across groups, with night and weekend shifts reported by more than 80% of respondents. High workload and inadequate work–life balance were also frequently mentioned in this context.

### Compatibility of family and career

Overall, 50% of participants reported having children (Table 2). On average, respondents had 1.85 children (95% confidence interval: 1.77–1.93; range: 1–5; SD: 0.039). However, significant differences were observed based on workplace setting. Among those working in outpatient care, 82.6% reported having children, compared to 50.9% of respondents from non-university departments and 34.7% from university departments (*p* < 0.001). Of participants without children, 82.2% expressed a desire to become parents in the future.

Most female respondents reported having their first child either toward the end of or after completing their specialty training. Female physicians employed at university departments were significantly more likely to become mothers after attaining specialist status (*p* = 0.003) and returned to work sooner after parental leave compared to their peers in non-university departments (*p* < 0.001). Specifically, 73.9% of parents at university departments resumed work within 12 months of childbirth, compared to 54.2% at non-university departments. When asked about their personal preference, 72.6% of all participants indicated a desire to return to work within 1 year following the birth of a child (Table 2). This preference was more frequently expressed by respondents at university departments (64.6%), compared to those in non-university departments (57.9%) and outpatient care (41.7%) (*p* < 0.001).

Across all workplace types, the majority of participants reported reducing their working hours upon returning from parental leave. This applied to 78.0% of respondents from university departments and 68.4% from non-university departments, though the difference was not statistically significant (*p* = 0.14; Table 4). A substantial majority (90.7%) indicated that their current workplace does not offer a structured reintegration program following parental leave (Table 3), while 79.0% expressed a wish for such a program to be implemented.

**Fig. 3:**
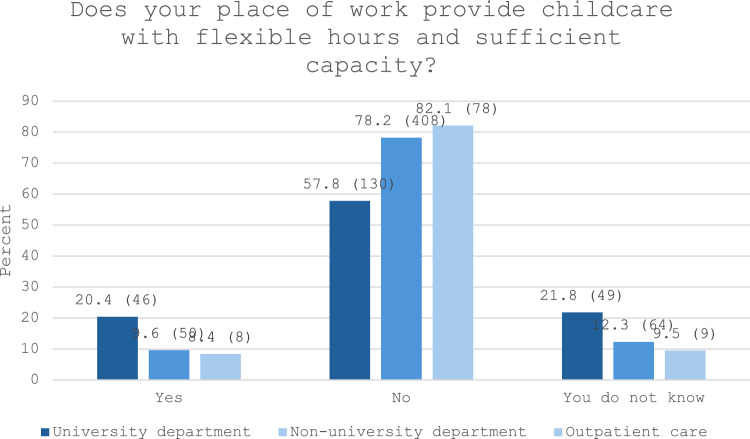
Childcare provided by employer in the university, non-university and outpatient care setting

In terms of workplace support, 20.4% of respondents employed at university departments reported sufficient flexibility and availability of childcare, compared to 9.6% at non-university departments and 8.4% in outpatient care (see Fig. 3).

Respondents employed at university departments were significantly more likely to report that their male colleagues took parental leave compared to those working in non-university departments (75.1 vs. 43.3%, *p* < 0.001). Overall, 50.4% of participants indicated that their employer actively supports parental leave. This proportion was highest among respondents from non-university departments (54.2%), followed by those from university departments (46.6%) and outpatient care settings (39.4%) (*p* = 0.037).

### Working-time models in different workplaces

Overall, 52.4% of respondents reported working full time (Table 4), with substantial variation across different workplace settings. Full-time employment was most common among those working in university departments (76.7%), compared to 47.1% in non-university departments and 25.0% in outpatient care. With regard to personal preferences, the desire to work full time was also highest among respondents employed at university departments (18.6%), followed by those in non-university departments (9.6%) and outpatient care settings (4.6%).

When asked which components of specialty training are inadequately addressed due to family leave or part-time employment, the most frequently cited area was surgical training (72.9 and 68.9%, respectively), followed by subspecialty consultations, including breast diagnostics and colposcopy (37.7 and 37.2%, respectively). In contrast, over 90% of participants indicated that training in emergency management, inpatient care, and conservative treatment modalities can be adequately maintained despite periods of parental leave or part-time work (Table 5).

## Discussion

The FARBEN study represents the first trinational investigation into the current status and individual preferences concerning work, career, and family models within the field of gynecology and obstetrics. This national sub-analysis specifically explored differences between university departments, non-university departments, and outpatient care settings—marking the first study of its kind to conduct such a comparison.

Significant differences were observed in gender distribution across workplace settings. Over 90% of residents in gynecology and obstetrics were female, and approximately one-third of the respondents from non-university departments reported not having a single male colleague among their resident cohort. In contrast, leadership positions such as heads of department were predominantly held by men, although the proportion of female department heads was higher in university departments compared to non-university institutions.

Historically, medical teams have been male-dominated. However, over the past 30 years, societal shifts have led to a marked increase in the proportion of women entering the medical profession [11–13]. Currently, approximately 75% of students enrolled in German medical schools are female, a trend that is increasingly reflected in the gender composition of physicians across both inpatient and outpatient care [14]. Within gynecology and obstetrics, the proportion of female practitioners is particularly high in non-university departments, while the gender distribution appears more balanced in university settings. Whether these discrepancies reflect differing career aspirations remains to be elucidated. As expected, respondents employed at university departments were significantly more likely to pursue an academic career. Male physicians more frequently reported aspirations to attain leadership roles and higher academic qualifications such as habilitation, suggesting that their preference for working in university settings may align with these career goals. However, female physicians at university departments also commonly pursue academic advancement, with one-third of the female respondents indicating an intention to complete a habilitation.

Gender role models may also influence career path decisions [15]. Particularly during crises such as the COVID-19 pandemic—when government-imposed restrictions significantly limited access to professional childcare and disrupted school attendance—a survey conducted at nine German university hospitals found that female physicians disproportionately assumed responsibility for family care and childcare compared to their male counterparts [15]. Consequently, a higher proportion of female respondents reported that they perceived their professional development to be at risk. This imbalance is further supported by a survey from the Marburger Bund—the largest professional association and trade union for physicians in Europe—where 41% of female physicians reported that societal conditions could hinder their career advancement [16]. However, in our study, engagement in scientific-academic training and education was strongly associated with the place of employment, irrespective of gender.

Notably, female physicians employed at university departments tended to become mothers at a later stage—most often toward the end of residency or as specialists—while those in non-university departments and outpatient settings more frequently became mothers during the middle of residency. Furthermore, female university-affiliated physicians returned to work sooner after childbirth. Two potential factors may explain this phenomenon. First, survey participants from university departments more frequently reported access to flexible childcare provided by their employer. It is, therefore, plausible that such support plays a crucial role in shaping decisions regarding the length of parental leave. Second, concerns about professional setbacks due to pregnancy and parental leave may influence family planning decisions. Milestones such as the completion of a doctoral degree, habilitation, or specialist training may be consciously prioritized before starting a family, particularly in more hierarchical environments such as university departments. This aligns with the finding that two–thirds of the respondents perceived surgical training to be negatively impacted by pregnancy or family leave.

New policies such as “Safe Surgery During Pregnancy” are promising steps toward improving working conditions and mitigating the negative impact of family planning on medical training [17, 18]. Importantly, not only physicians but also female medical students express concerns about potential professional disadvantages resulting from pregnancy [14]. As such, the implementation of structured reintegration programs—such as dedicated mentorships and family-compatible surgical training pathways—after parental leave is essential. These programs could include supervised re-entry into operative training by colleagues with similar prior experiences. International models from countries such as Denmark, the Netherlands, and Switzerland may serve as exemplary frameworks for improving the current training infrastructure [19].

A good work–life balance appears to be a decisive factor in the selection of a medical specialty and may influence career decisions as early as the initial years of medical school [14]. Our study confirms that the desire to successfully combine a professional career with private and family life is a key priority for physicians across all employment settings—university departments, non-university departments, and outpatient care. These findings align with existing international data indicating that flexibility in the workplace is a widely desired attribute among physicians [20, 21]. Childcare, in particular, remains a critical concern for many [12]. Inflexible childcare arrangements—especially in kindergartens—pose significant challenges for parents working shifts, such as hospital staff. It is, therefore, reasonable to assume that expanding access to flexible and adequate childcare options for healthcare workers could enhance employee satisfaction and may encourage a greater number of physicians to return to work full time or increase their working hours after starting a family.

Although university departments generally reported a better availability of employer-provided childcare, nearly 80% of university-employed respondents indicated that childcare options with flexible hours and sufficient capacity were not available. In addition to childcare, flexible working conditions were also highlighted as a major concern. Only one in eight respondents preferred to work full time in the future, with this preference being twice as common among university-employed physicians compared to those from non-university departments. Autonomy and flexibility in scheduling can significantly enhance job satisfaction allow better adaptation to individual life circumstances, reduce rates of absenteeism, and lower the likelihood of premature retirement [7, 22, 23]. Moreover, in the context of growing physician shortages, improving working conditions could have a substantial impact not only on healthcare delivery but also on economic outcomes. Several studies have demonstrated that imposing a limit on the maximum number of weekly working hours—excluding overtime—can contribute to increased job satisfaction [24, 25].

### Strengths and limitations

The FARBEN study represents the largest investigation to date in German-speaking countries concerning working models, family life, and work–life balance in the field of gynecology and obstetrics. For the first time, this study provides a differentiated analysis of the workplace-specific conditions in university departments, non-university departments, and outpatient care settings. The study was initiated and supported by the young forums of the national gynecological societies, which likely explains the high proportion of participants currently in specialty training—approximately two–thirds of the total sample. Consequently, the findings may not be fully generalizable to the entire population of gynecologists and obstetricians.

It is also important to note that specialty training in gynecology and obstetrics in Germany differs substantially from that in many non-German-speaking countries, where mandatory rotations across different hospitals are common during residency. This is not the case in Germany. While the distribution of respondents between university and non-university departments reflects the current national structure, the outpatient care setting was underrepresented, with only 10.8% of participants working in this area.

Although outpatient care plays a less prominent role in residency training, more than two–thirds of the physicians ultimately transition into this setting after completing their training. Due to the small sample size from outpatient care in this study, no definitive conclusions can be drawn for this subgroup. Future research focusing on this majority group of physicians is warranted. In particular, studies examining workload and the compatibility of family and career in the outpatient setting could yield valuable insights and help clarify why so many physicians leave hospital-based roles after completing residency.

A key focus of this study was to highlight gender-related disparities in the context of workplace characteristics, family compatibility, and academic career aspirations. However, other sociopolitical and intersectional factors—such as socioeconomic status and migration background—were not captured in the FARBEN study. These dimensions warrant further investigation in future research to provide a more comprehensive understanding of career trajectories and challenges in the field of gynecology and obstetrics.

## Conclusions

The compatibility of family life and career, as well as a good work–life balance, appears to be of central importance to the majority of residents in gynecology and obstetrics, irrespective of their workplace, and strongly influences their choice of specialty. While university departments are sometimes perceived as less family-friendly than non-university settings, this sub-analysis of the FARBEN study indicates that the availability and flexibility of employer-provided childcare are, in fact, more favorable in university departments. Furthermore, respondents employed in university settings were more likely to work under a female head of department compared to those in non-university settings.

This study highlights several challenges young physicians may encounter in the context of family planning. In particular, pregnancy and parental leave were frequently cited as negatively impacting surgical training. Moreover, two–thirds of the survey participants expressed the intention to return to work within 1 year of taking parental leave. Consequently, it is imperative that employers foster an environment that enables physicians to balance their personal and professional lives according to their individual needs. In this context, expanding the infrastructure for childcare at the workplace is essential. In addition, structured reintegration programs—such as targeted mentorships or family-compatible surgical training pathways—should be implemented to support re-entry after parental leave. Existing models from countries such as Denmark, the Netherlands, and Switzerland could serve as valuable references.

Despite evolving social conditions, the present analysis demonstrates that two out of five physicians employed at university departments aspire to a scientific-academic career. In light of ongoing workforce shortages, further support programs are urgently needed. In particular, expanding family-friendly working conditions for women is crucial to enable the successful combination of academic career development and family life.

## Data Availability

No datasets were generated or analysed during the current study.

## Appendix

Questionnaire, including all questions directed to participants from all nations.

**Table:**

1.** What is your sex?** Female Male Diverse
2. **How old are you?** 18–20 21–30 31–40 41–50 51–60 61–70 71 or older
3. **What is your current further education/professional position?** Student Students in their practical year Resident 1 st year Resident 2nd year Resident 3rd year Resident 4th year Resident 5th year Resident 6th year Resident > 6th year Specialist physician Senior physician Chief physician Gynecologist in outpatient care
4. **Your professional goal is this position:** Chief physician (Leading) senior physician Employed medical specialist in the department Employed in outpatient care Self-employed in outpatient care Other (please specify)
5. **Why would you not like to work in the department long term? (multiple answers possible**) Too much responsibility Too high workload Too little work/life balance Insufficient compatibility of family/work Night and weekend shifts Duties in the delivery room Financially unattractive Inflexible working hours
6. **In which country do you work or study?** Germany Austria Switzerland
7. **Do you have children?** Yes No
8. **How many children do you have?**
9. **How old were you when you had your first child?**
10. **With your first child you were:** Student Students in their practical year Resident 1 st year Resident 2nd year Resident 3rd year Resident 4th year Resident 5th year Resident 6th year Resident > 6th year Specialist physician Senior physician Chief physician Gynecologist in outpatient care
11. **How long did you not work after giving birth? (For multiple children with different time off, please use multiple answers)** 2 years per child 1–2 years per child 9–12 months per child 6–8 months per child 3–5 months per child < 3 months per child You did not take any family leave Other option (please specify)
12. **Why did you, as a parent, not take any family leave? (Multiple answers possible)** Not necessary/not desired due to family situation Fear of professional disadvantage Incompatible with career plans Financial obligations Other reasons (please specify)
13. **Do you want to become a parent in the future?** Yes No You are not sure yet
14. **How long would you like to pause work after the birth of your child as a parent?** 2 years per child 1–2 years per child 9–12 months per child 6–8 months per child 3–5 months per child < 3 months per child You do not want to take any family leave You are not sure yet
15. **Your workplace** University department Non-university women’s department with a total of > 25 physicians Non-university women’s department with a total of 15–25 physicians Non-university women’s department with a total of < 15 physicians Outpatient care/MVZ (employed) Outpatient care/MVZ (self-employed) Student Other (Combination of outpatient and department, outpatient care in Austria…)
16. **How many male resident physicians are there in your team?** > 50% Ca. 50% Ca. 40% Ca. 30% Ca. 20% Ca. 10% There is no male resident doctor
17. **How many male senior physicians are there in the team?** 100% 80– < 100% 60– < 80% 40– < 60% 20– < 40% 1– < 20% There is no male senior physician
18. **Your head of department is** Female Male Diverse Other (position not filled, dual leadership, etc.)
19. **How many part-time positions do you think a team of residencies can handle?** 0% 1–20% > 20–40% > 40–60% > 60–80% > 80–99% 100%
20. **How many part-time positions do you think a team of senior physicians can handle?** 0% 1–20% > 20–40% > 40–60% > 60–80% > 80–99% 100%
21. **What percentage of mothers in your department takes family leave?** 100- > 80% > 60–80% > 40–60% > 20–40% 1–20% Mothers do not take family leave at your department You do not know There are no mothers at your department
22. **What percentage of fathers in your department take family leave?** 100– > 80% > 60–80% > 40–60% > 20–40% 1–20% Fathers do not take family leave at your department You do not know There are no fathers at your department
23. **How long does it take for fathers to return to work in your department after family leave?** Usually the same as for mothers Usually shorter than for mothers Usually longer than for mothers
24. **The use of family leave for women leads to disadvantages in further medical training/professional careers:** Do not agree at all Largely disagree Rather disagree Neither Rather agree Largely agree Strongly agree
25. **The use of family leave for men leads to disadvantages in further medical training/careers:** Do not agree at all Largely disagree Rather disagree Neither Rather agree Largely agree Strongly agree
26. **Family leave at your workplace is supported:** Do not agree at all Largely disagree Rather disagree Neither Rather agree Largely agree Strongly agree
27. **In your opinion, after what period of time does a ‘career break’ occur for parents on family leave?** From 3 to 6 months From 6 to 9 months From 9 to 12 months From 1 to 2 years From > 2 years Family leave does not cause a career break Family leave causes a career break regardless of the duration
28. **In your opinion, what percentage of part-time work results in a ‘career break’?** From 90% From 80% From 70% From 60% From 50% From < 50% Part-time does not cause a career setback Part-time causes a career setback regardless of the percentage share
29. **Working part-time is at the expense of your own medical training/professional career** Do not agree at all Largely disagree Rather disagree Neither Rather agree Largely agree Strongly agree
30. **Your preferred working time model is:** 100% 90–99% 80–89% 70–79% 60–69% 50–59% < 50%
31. **Your current working time model is:** 100% 90–99% 80–89% 70–79% 60–69% 50–59% < 50% You are currently on maternity leave You are currently on family leave as a parent You are currently looking for work/not yet employed
32. **How many days do you work per week (not including on-call duty)?** 1 2 3 4 5
33. **What are your working hours on the days you work?** Full days Shorter daily working hours A mixture of full and shorter days
34. **Did you reduce your working hours after the birth of your child?** I have no children Yes No
35. **How long have you been working part-time?** Since the 1 st year of residency Since the 2nd year of residency Since the 3rd year of residency Since the 4th year of residency Since the 5th year of residency Since the 6th year of residency Since the > 6th year of residency Since you have been a medical specialist Since you have been a senior physician Since you have been a head physician Since you have been practicing as a gynecologist in outpatient care
36. **To what extent do you agree with this statement: extending your training time by working part-time is a problem for you:** Do not agree at all Largely disagree Rather disagree Neither Rather agree Largely agree Strongly agree
37. **Your reasons for working part-time are (multiple answers possible)** Caring for children Caring for relatives Desire for a better work–life balance Too extensive workload Time needed for academic work Other: (please specify)
38. **How many hours/week do you spend caring for relatives?** < 5 h/week 5–10 h/week 11–15 h/week > 16 h/week
39. **Part-time workers lead to an increased burden on the team:** Yes, regardless of fixed days off or reduced daily hours Yes, regardless of fixed days off or reduced daily hours Yes, but only with reduced daily working hours Yes, but only with fixed days off No, not at all
40. **What solutions would you propose for avoiding a possible personnel burden due to part-time workers? (multiple answers possible)** ^2)^ Fixed job sharing Fixed job sharing (2 physicians each 50% with duty splitting) Part-time employees work exclusively full days with fixed days off I do not know Other (please specify)
41. **The optimal time to become a parent is for you:** During medical school During specialty training As medical specialist As senior (leading) physician As chief physician As a physician in outpatient care There is no optimal time
42. **Can you imagine taking a sabbatical?** You do not want to take a sabbatical Maximum 3 months 3–6 months 6–12 months More than 12 months
43. **A sabbatical at your workplace is supported:** Do not agree at all Largely disagree Rather disagree Neither Rather agree Largely agree Strongly agree
44. **To what extent do you agree with this statement: you support the decision of your colleagues who have decided to take a sabbatical** Do not agree at all Largely disagree Rather disagree Neither Rather agree Largely agree Strongly agree
45. **Does your workplace have a concept for returning to work after a longer period of family leave (e.g., return to work with an initially reduced workload)?** Yes, a re-entry concept is available No, I am not aware of the existence of such a concept. But I think it is desirable No, I am not aware of the existence of such a concept and do not consider it necessary
46. **What reintegration opportunities does your concept provide?** **Free-text answer**
47. **Does your place of work provide childcare with flexible hours and sufficient capacity?** Yes No You do not know
48. **Would childcare close to the workplace be a factor when choosing an employer?** Yes, this would influence my choice of employer No, it does not matter
49. **Your highest academic title is:** Master’s degree doctorate Habilitation professorship You do not have an academic title
50. **You would like to achieve habilitation:** Yes No You do not know yet
51. **In your opinion, which parts of further training in gynecology and obstetrics cannot be adequately implemented due to family leave (multiple answers possible)?** Obstetrics training Acquisition of special skills in gynecological diagnostics (e.g., breast sonography and dysplasia) Surgical training Care of inpatients Conservative management of gynecological clinical diseases Management of gynecological emergencies All parts of the training are adequately implemented despite family leave
52. **In your opinion, which parts of specialty training in gynecology and obstetrics cannot be adequately implemented due to part-time work(multiple answers possible)** Obstetric training Acquisition of special skills in gynecological diagnostics (e.g., breast sonography, dysplasia) Surgical training Care of inpatients Conservative management of gynecological diseases Management of gynecological emergencies All parts of the training are adequately implemented despite part-time work
53. **Are gynecologists in specialty training disadvantaged in their professional career because of gender?** Do not agree at all Largely disagree Rather disagree Neither Rather agree Largely agree Strongly agree
54. **What, in your opinion, would need to change in gynecology to ensure that no one feels disadvantaged in their professional life because of their gender?** ** Free-text answer**
